# Supplementary material for: Resolving the full spectrum of human genome variation using Linked-Reads
Source: Genome Res. 2019 Apr;29(4):635–45. doi: 10.1101/gr.234443.118 (PMC6442396; doi:10.1101/gr.234443.118)
Supplement: Supplemental Material [file supp_gr.234443.118_Supplemental_longranger-2.2.zip › longranger-2.2.tar.bz2!/lr2.2-public/lib/stan/cmdstan/stan/doc/api/html/classstan_1_1_double_vector_view.html]

Stan: stan::DoubleVectorView< used, is\_vec > Class Template Reference


|  |  |
| --- | --- |
|  | Stan  2.5.0  probability, sampling & optimization |


- Main Page
- Related Pages
- Namespaces
- Classes
- Files

- Class List
- Class Index
- Class Hierarchy
- Class Members

All Classes Namespaces Files Functions Variables Typedefs Enumerations Enumerator Friends Macros Pages

- stan
- DoubleVectorView

Public Member Functions |
List of all members

stan::DoubleVectorView< used, is\_vec > Class Template Reference

DoubleVectorView allocates double values to be used as intermediate values.
More...

`#include <traits.hpp>`

|  |  |
| --- | --- |
| Public Member Functions | |
|  | DoubleVectorView (size\_t) |
|  | |
| double & | operator[] (size\_t) |
|  | |

## Detailed Description

### template<bool used, bool is\_vec> class stan::DoubleVectorView< used, is\_vec >

DoubleVectorView allocates double values to be used as intermediate values.

There are 2 template parameters:

- used: boolean variable indicating whether this instance is used. If this is false, there is no storage allocated and operator[] throws.
- is\_vec: boolean variable indicating whether this instance should allocate a vector, if it is used. If this is false, the instance will only allocate a single double value. If this is true, it will allocate the number requested.

These values are mutable.

Definition at line 358 of file traits.hpp.

## Constructor & Destructor Documentation

template<bool used, bool is\_vec>

|  |  |  |  |  |  |
| --- | --- | --- | --- | --- | --- |
| stan::DoubleVectorView< used, is\_vec >::DoubleVectorView | ( | size\_t |  | ) |  |

inline

Definition at line 360 of file traits.hpp.

## Member Function Documentation

template<bool used, bool is\_vec>

|  |  |  |  |  |  |
| --- | --- | --- | --- | --- | --- |
| double& stan::DoubleVectorView< used, is\_vec >::operator[] | ( | size\_t |  | ) |  |

inline

Definition at line 361 of file traits.hpp.

---

The documentation for this class was generated from the following file:

- src/stan/meta/traits.hpp


---

[ Stan Home Page ]

*© 2011–2014,
Stan Development Team.*
